# Cognitive Stimulation and Training in People with Mild-to-Moderate Alzheimer’s Disease: A Scoping Review

**DOI:** 10.3390/brainsci16090944

**Published:** 2026-09-03

**Authors:** Rosa Martins, Nélia Carvalho, Ricardo Loureiro, Joana Bernardo Loureiro

**Affiliations:** 1 Piaget Institute, Viseu, Estrada do Alto do Gaio, 3515-771 Lordosa, Portugal; rmartins.viseu@gmail.com; 2 ULS Viseu Dão-Lafões, USF Montemuro, Castro Daire, 3600-180 Viseu, Portugal; mncarvalho2@ulsvdl.min-saude.pt; 3 Center for Innovative Care and Health Technology (ciTechCare), School of Health Sciences, Polytechnic of Leiria, 2411-901 Leiria, Portugal; ricardo.loureiro@ulo.pt

**Keywords:** Alzheimer’s disease, cognitive stimulation, cognitive training, reminiscence therapy, virtual reality, computerized cognitive training, scoping review

## Abstract

**Highlights:**

**What are the main findings?**

- The included studies showed short-term, outcome-specific patterns across structured cognitive stimulation and training interventions.
- Technology-assisted and conventional cognitive training primarily showed signals in cognitive outcomes, whereas reminiscence-based interventions more consistently reported changes in depressive and neuropsychiatric symptoms.

**What are the implications of the main findings?**

- Cognitive stimulation and training may complement comprehensive, person-centred care, but current evidence is insufficient to establish comparative effectiveness or long-term benefits.
- Larger studies with standardized outcomes, replicable interventions, and longer follow-up are needed, including evaluation of maintenance or booster strategies.

**Abstract:**

Background/Objectives: Cognitive stimulation and cognitive training are used as non-pharmacological approaches to support people living with Alzheimer’s disease, but Alzheimer-specific evidence is dispersed across heterogeneous intervention formats. This scoping review mapped structured cognitive stimulation and training interventions evaluated in people with mild-to-moderate Alzheimer’s disease and summarized the cognitive, emotional, functional, and follow-up outcomes reported. Methods: The review was conducted using the Joanna Briggs Institute methodology and reported according to PRISMA-ScR. PubMed, SciELO, PEDro, LILACS, and Google Scholar were searched on 24 February 2025 for studies published between January 2015 and 24 February 2025 in English, Portuguese, or Spanish. Intervention studies were eligible. Two reviewers independently screened records and charted data, with disagreements resolved by a third reviewer. Results: Of 352 records identified, five studies involving 245 participants were included. Interventions comprised virtual-reality cognitive stimulation, conventional cognitive training, group reminiscence therapy, a multicomponent music–reminiscence–reality-orientation intervention, and computerized cognitive training. In the limited technology-assisted evidence, statistically significant changes were reported in global cognition or selected memory, language, attention, and executive outcomes. Conventional cognitive training showed signals of improvement in initiative and temporary stabilization of memory, whereas reminiscence-based interventions primarily reported changes in depressive and neuropsychiatric symptoms. Where longer follow-up was available, benefits diminished over time. Conclusions: The mapped evidence suggests that structured cognitive stimulation and training may produce short-term, outcome-specific benefits in mild-to-moderate Alzheimer’s disease. Given the small and heterogeneous evidence base, these findings represent modality-related patterns within the included studies and should not be interpreted as evidence of comparative effectiveness.

## 1. Introduction

Dementia is a major cause of disability and dependency in later life. The World Health Organization estimates that 57 million people were living with dementia in 2021 and that Alzheimer’s disease accounts for approximately 60–70% of cases [1]. Although pharmacological treatment is an important component of clinical management, comprehensive dementia care also includes non-pharmacological strategies intended to support cognition, daily functioning, participation, and quality of life [1,2].

Cognitive stimulation commonly involves a range of engaging activities designed to provide general stimulation for thinking, concentration, and memory, frequently in a social context [2]. Cognitive training is more task-specific and generally consists of guided practice on standardized tasks targeting particular cognitive domains [3]. These approaches differ conceptually from cognitive rehabilitation, which is individualized and oriented toward personally meaningful functional goals [3,4]. In practice, however, the terminology and content of interventions often overlap, particularly in studies that combine memory exercises, reminiscence, reality orientation, music, computerized tasks, or simulations of everyday activities.

A recent Cochrane review of cognitive stimulation in people with dementia found small probable benefits for cognition and possible benefits in other outcomes, while also emphasizing substantial heterogeneity in intervention delivery, frequency, and participant characteristics [2]. Alzheimer-specific studies are especially heterogeneous, ranging from conventional group interventions to computerized training and immersive virtual-reality environments. Mapping these modalities separately in people with a confirmed Alzheimer’s disease diagnosis may clarify what has been evaluated, which outcomes have been reported, and where important evidence gaps remain.

This scoping review therefore aimed to map structured cognitive stimulation and cognitive training interventions evaluated in adults with mild-to-moderate Alzheimer’s disease. The review question was: What structured cognitive stimulation or training interventions have been evaluated in people with mild-to-moderate Alzheimer’s disease, and what cognitive, emotional, functional, feasibility, and follow-up outcomes have been reported?

## 2. Materials and Methods

### 2.1. Design and Reporting

This scoping review was conducted in accordance with the Joanna Briggs Institute methodological guidance for scoping reviews [5] and is reported according to the Preferred Reporting Items for Systematic Reviews and Meta-Analyses extension for Scoping Reviews (PRISMA-ScR) [6]. The completed PRISMA-ScR checklist is provided as Supplementary Material S1. A protocol for this scoping review was not prospectively registered or published.

### 2.2. Eligibility Criteria

Eligibility criteria were organized using the Population–Concept–Context framework.

Population. Studies were eligible when participants were adults with a clinical diagnosis of mild-to-moderate Alzheimer’s disease. Studies involving mixed dementia populations were eligible only when Alzheimer-specific data were reported separately. Studies of cognitively healthy participants, people considered only at risk of Alzheimer’s disease, mild cognitive impairment without a confirmed Alzheimer’s disease diagnosis, severe or advanced Alzheimer’s disease, or other forms of dementia without separately reported Alzheimer-specific data were excluded.

Concept. Eligible studies evaluated a structured, repeated, non-pharmacological intervention whose main purpose was to stimulate, train, or maintain cognitive functioning. Eligible modalities included conventional cognitive stimulation or training, reminiscence therapy, reality orientation, computerized cognitive training, and virtual-reality-based cognitive stimulation. Multicomponent interventions were eligible when a cognitively oriented component was central to the intervention. Pharmacological interventions, diagnostic tools, caregiver-only education, and interventions based exclusively on physical exercise, yoga, sensory stimulation, social robotics, or music without an explicit cognitive or reminiscence component were excluded. The restriction to controlled intervention studies was intended to provide a more comparable basis for mapping reported intervention outcomes and was not intended to support causal-effectiveness conclusions. Consequently, uncontrolled feasibility and implementation evidence may not be represented in this review.

Context. Studies conducted in home, community, day-care, outpatient, hospital, nursing-home, or residential-care settings were eligible.

Types of evidence sources. Randomized controlled trials, non-randomized controlled studies, and controlled pilot intervention studies published as full-text peer-reviewed articles between January 2015 and February 2025 were considered. Publications in English, Portuguese, or Spanish were eligible. Reviews, protocols, editorials, opinion papers, conference abstracts, case reports, uncontrolled case series, and observational studies without an intervention were excluded.

The lower publication-date limit of January 2015 was selected to focus the review on contemporary intervention research and to capture the period in which technology-assisted approaches, including computerized training and virtual reality, became increasingly prominent in Alzheimer’s disease care. The upper limit corresponded to the date of the final search, 24 February 2025. English, Portuguese, and Spanish were selected because these were the languages in which the review team could reliably assess full-text publications without translation. This pragmatic language restriction may have resulted in the exclusion of relevant studies published in other languages and was therefore considered a limitation of the review.

### 2.3. Information Sources and Search

The search was conducted on 24 February 2025 in PubMed, SciELO, LILACS, PEDro, and Google Scholar.

The Google Scholar search retrieved 100 records, all of which were included in the screening set. No additional prioritization, relevance-based selection, or truncation criterion was applied.

The following database-specific search strings were used exactly as reported in the Table 1.

The searches covered publications from January 2015 to February 2025. Publications in English, Portuguese, or Spanish were considered during eligibility assessment. Google Scholar contributed 100 records to the screening set. Records were managed in Mendeley^®^ (Mendeley Reference Manager v2.145.0), and duplicate records were removed before screening.

### 2.4. Selection of Evidence Sources

**Figure 1:**
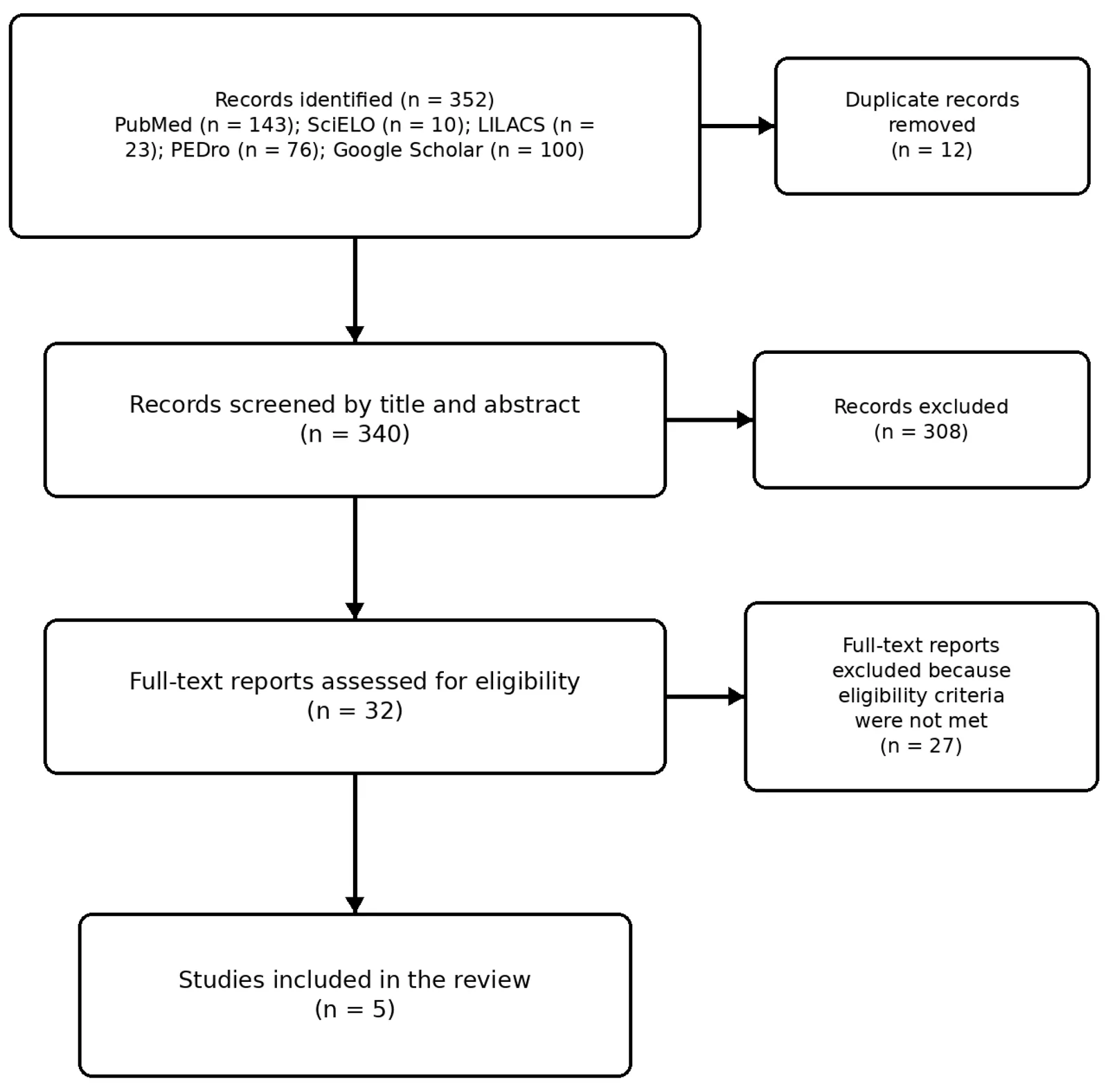
PRISMA-ScR flow diagram of study selection.

Two reviewers independently screened titles and abstracts against the eligibility criteria. Potentially relevant reports were then assessed in full text. Disagreements were resolved through discussion and, when required, consultation with a third reviewer. The selection process is summarized in Figure 1.

### 2.5. Data Charting and Synthesis

A standardized data-charting form was used to extract the author, year, country, study design, setting, participant characteristics, intervention and comparator, intervention dose, outcome domains, main findings, follow-up, and limitations. Data were summarized descriptively in tables and through a narrative synthesis organized by intervention modality and outcome domain. Two reviewers independently charted the data using a standardized data-charting form. Disagreements were resolved through discussion and, when required, consultation with a third reviewer.

Consistent with the mapping purpose of this scoping review, no formal risk-of-bias or certainty-of-evidence assessment was undertaken.

Accordingly, the findings are interpreted as a descriptive mapping of reported interventions and outcomes and not as estimates of intervention effectiveness or certainty of evidence.

## 3. Results

### 3.1. Study Selection

The search identified 352 records: PubMed (*n* = 143), SciELO (*n* = 10), LILACS (*n* = 23), PEDro (*n* = 76), and Google Scholar (*n* = 100). After the removal of 12 duplicates, 340 records were screened by title and abstract, and 308 were excluded. Thirty-two full-text reports were assessed for eligibility. Twenty-seven reports were excluded for the following primary reasons: ineligible population (*n* = 10), intervention not meeting the predefined cognitive stimulation or cognitive training concept (*n* = 5), ineligible study design or publication type (*n* = 5), and Alzheimer-specific data not reported separately (*n* = 7). Finally, five studies were included in the final review. The flow of study selection is presented in Figure 1.

### 3.2. Characteristics of the Included Studies

Six reports describing five distinct studies were published between 2016 and 2021 and were conducted in Portugal, Italy, China, and Spain [7,8,9,10,11,12]. Together, the five studies included 245 participants with mild-to-moderate or early-stage Alzheimer’s disease. Four studies used randomized controlled designs, and one used a controlled quasi-experimental pilot design. One randomized trial was reported in an original publication and a subsequent 12-month follow-up report. Intervention duration ranged from eight weeks to three months, and the longest reported follow-up was 12 months. Characteristics of the included studies are presented in the Table 2.

### 3.3. Intervention Modalities and Reported Outcomes

The included interventions represented five specific intervention types: technology-assisted cognitive stimulation [7], conventional cognitive training [8], group reminiscence therapy [9], a multicomponent music–reminiscence–reality-orientation intervention [10], and technology-assisted computerized cognitive training [11,12]. For synthesis purposes, these were subsequently organized into three broader modalities: technology-assisted cognitive interventions, conventional cognitive training, and reminiscence-based or multicomponent interventions. The reported outcomes and limitations are summarized in the Table 3.

#### 3.3.1. Technology-Assisted Cognitive Interventions

Two studies, reported in three publications, evaluated technology-assisted approaches [7,11,12]. Oliveira et al. used a technology-assisted virtual-reality intervention based on simulated instrumental activities of daily living and reported a statistically significant change in global cognition, although significant effects were not consistently observed across the other cognitive and functional outcomes assessed [7]. Cavallo et al. reported the immediate and six-month outcomes of the computerized intervention, while Cavallo and Angilletta subsequently reported the 12-month follow-up findings [11,12]. Together, these studies indicate the potential of digital formats while also highlighting small samples, uncertain transfer to everyday functioning, and limited durability.

#### 3.3.2. Conventional Cognitive Training

Giovagnoli et al. compared cognitive training with active music therapy and neuroeducation [8]. Cognitive training improved initiative and temporarily stabilized episodic memory, whereas the two non-cognitive comparators produced larger psychosocial changes. The decline observed across groups at follow-up suggests that maintenance or booster sessions may be relevant, although the study was not designed to establish an optimal maintenance schedule.

#### 3.3.3. Reminiscence-Based and Multicomponent Interventions

Li et al. evaluated structured group reminiscence therapy and reported benefits for depressive and neuropsychiatric symptoms [9]. Onieva-Zafra et al. combined familiar music, reminiscence, and reality orientation and found a reduction in depression in a small nursing-home sample [10]. In these studies, the clearest signals concerned emotional and behavioural outcomes rather than consistent improvement across cognitive or functional domains.

## 4. Discussion

This scoping review mapped five studies evaluating structured cognitive stimulation or cognitive training interventions in people with mild-to-moderate Alzheimer’s disease. The identified interventions included conventional cognitive training, group reminiscence therapy, a multicomponent music–reminiscence–reality-orientation intervention, computerized cognitive training, and immersive virtual reality. Overall, the findings were heterogeneous and outcome-specific. Technology-assisted and conventional cognitive training interventions primarily reported changes in cognitive outcomes, whereas reminiscence-based interventions more consistently affected depressive or neuropsychiatric symptoms. Evidence concerning functional transfer, acceptability, and long-term maintenance remained limited.

The pattern identified in this review is broadly consistent with the wider dementia literature. A recent systematic review and meta-analysis by Paggetti et al. found that both group and individual cognitive stimulation may support global cognitive functioning in people living with dementia, while cognitive training appeared particularly beneficial among people with mild dementia [13]. However, that review also identified substantial heterogeneity in intervention modality, intensity, duration, delivery format, participant characteristics, and outcome measurement. The certainty of evidence for cognitive training was lower than that for cognitive stimulation, and improvements in trained tasks did not consistently translate into everyday functioning [13]. Although this broader review was not restricted to Alzheimer’s disease, its findings reinforce the need to distinguish cognitive stimulation, cognitive training, and cognitive rehabilitation rather than treating them as interchangeable interventions.

More recent Alzheimer-specific evidence also supports the potential value of structured cognitive stimulation as an adjunct to pharmacological treatment. In an evaluator-blinded randomized controlled trial published after the search conducted for the present review, Cai et al. compared 14 weekly sessions of modified cognitive stimulation therapy combined with standard pharmacotherapy against pharmacotherapy alone in 80 people with mild-to-moderate Alzheimer’s disease [14]. The intervention was associated with better post-intervention cognitive performance, activities of daily living, and quality of life. The adjusted between-group differences favoured the cognitive stimulation group for ADAS-Cog, ADL, and QOL-AD outcomes [14]. Although this study falls outside the formal search period of the present review, it provides useful contextual evidence regarding the subsequent development of this field. Nevertheless, the absence of an active social-contact control, the relatively small sample, and the limited follow-up period mean that the specific contribution and durability of cognitive stimulation remain uncertain.

The different outcome profiles observed across intervention modalities may reflect differences in their therapeutic targets and mechanisms. Cognitive training generally involves repeated practice of specific cognitive operations, which may explain the improvements reported in memory, attention, language, or executive measures. By contrast, reminiscence-based interventions incorporate autobiographical memory, personal meaning, emotional expression, and social interaction. These characteristics may help explain why the clearest benefits in the reminiscence studies concerned depression and neuropsychiatric symptoms rather than consistent improvements across cognitive or functional outcomes. The wider evidence synthesized by Paggetti et al. similarly suggests that cognitive outcomes depend on the intervention modality, dementia severity, delivery format, and correspondence between the trained activities and the outcomes assessed [13].

Technology-assisted approaches may offer additional opportunities for personalization, repeated practice, ecological simulation, and delivery across different settings. In the present review, virtual reality was used to reproduce instrumental activities of daily living, whereas computerized cognitive training targeted memory, attention, executive functioning, and language [7,11,12]. However, technology should be understood as a mode of delivery rather than as evidence of superior effectiveness in itself. The clinical value of a digital intervention is likely to depend on whether it is accessible, meaningful, appropriately challenging, and integrated with professional guidance and interpersonal interaction.

More recent literature published outside the search period provides additional context for this interpretation. Fang et al. examined cognitive stimulation and digital cognitive training within a broader integrated care model comprising person-centred assessment, physical exercise, communication strategies, sensory interventions, digital technologies, and structured caregiver support [15]. Over 24 months, the integrated-care group demonstrated slower cognitive decline, better quality of life and functional outcomes, fewer behavioural symptoms, and reduced caregiver burden compared with usual care [15]. These findings suggest that cognitive interventions may be most clinically meaningful when integrated into comprehensive and person-centred care. However, because the intervention contained several coordinated components and used a quasi-experimental design, the observed outcomes cannot be attributed specifically to cognitive stimulation or digital training.

The implementation of digital and home-supported interventions also requires consideration of family caregivers. The systematic review and metasynthesis by Yu et al. showed that well-designed web-based psychoeducation programmes can enhance caregivers’ knowledge, self-efficacy, coping skills, and sense of empowerment [16]. Caregivers particularly valued relevant and stage-appropriate information, peer connection, professional facilitation, flexible access, and opportunities to revisit educational resources. Conversely, poor internet access, limited digital literacy, time constraints, information overload, and the absence of individual support impeded engagement [16]. Although this evidence does not demonstrate a direct effect on the cognition of people with Alzheimer’s disease, it identifies implementation conditions that may influence adherence, continuity, and the feasibility of home-based cognitive activities.

Caregiver involvement may be particularly relevant to intervention maintenance. In the studies mapped in this review, cognitive and initiative-related improvements diminished after treatment cessation, with deterioration observed at three months in the study by Giovagnoli et al. and attenuation of computerized-training gains at 12 months in the follow-up reported by Cavallo and Angilletta [8,12]. The wider meta-analytic evidence also highlights considerable variability in intervention frequency and intensity, with many studies using brief or low-intensity programmes [13]. Accordingly, future research should examine whether maintenance sessions, booster programmes, supervised home practice, or blended professional–caregiver delivery can sustain benefits beyond the immediate intervention period.

The findings also suggest that uniform intervention protocols may not be equally appropriate for all participants. Cai et al. identified educational attainment, baseline cognitive status, regular physical activity, and engagement in hobbies as exploratory predictors of response to cognitive stimulation therapy [14]. These results are compatible with the possible influence of cognitive reserve, lifestyle, and preserved learning capacities. Paggetti et al. similarly emphasized the potential contribution of educational, cultural, cognitive-reserve, and lifestyle characteristics to the variability in treatment response [13]. These findings should not be interpreted as indicating that people with lower education or more impaired cognition are unsuitable for cognitive intervention. Rather, they support adapting task complexity, pace, repetition, session duration, group size, sensory presentation, and the degree of caregiver or professional support to each person’s remaining abilities and preferences.

For nursing and multidisciplinary practice, the evidence supports the cautious inclusion of structured cognitive activities as an adjunct to comprehensive Alzheimer’s disease care. Intervention selection should be based on disease stage, cognitive and functional capacity, sensory and motor abilities, cultural background, digital familiarity, personal history, emotional response, and the care environment. Nurses may have a particularly important role in assessing these factors, tailoring activities, monitoring engagement and distress, educating caregivers, and coordinating cognitive interventions with pharmacological, functional, psychosocial, and behavioural care. Technology should augment rather than replace therapeutic relationships, and digital interventions should retain meaningful human guidance and social interaction [17,18,19,20].

Future trials should recruit larger and more diverse samples, use clearly described and replicable intervention protocols, and include active comparators capable of controlling for attention, expectation, group participation, and social contact. Core outcomes should encompass not only neuropsychological test performance but also everyday functioning, participation, quality of life, neuropsychiatric symptoms, caregiver outcomes, adherence, acceptability, adverse events, and implementation feasibility. Longer follow-up is required to determine whether short-term gains are maintained and to identify the optimal timing, frequency, intensity, and duration of maintenance or booster strategies.

This review has several limitations.

Although five information sources were searched, the search strategy was relatively focused and relied primarily on the term ‘cognitive stimulation’. Studies indexed exclusively under related concepts such as cognitive training, cognitive rehabilitation, computerized training, reminiscence, reality orientation, psychosocial intervention, or non-pharmacological intervention may therefore have been missed. In addition, databases such as CINAHL, PsycINFO, Embase, Cochrane CENTRAL, Scopus, and Web of Science were not searched.

The final search was conducted on 24 February 2025. Consequently, studies published subsequently were outside the formal evidence set of this review. More recent studies [14,15] discussed in this manuscript are used only to contextualize subsequent developments in the field and should not be interpreted as part of the scoping-review evidence base. Given the evolving literature in this field, a future update using a broader search strategy, additional multidisciplinary databases, and a more recent search date is warranted.

This review was restricted to peer-reviewed publications in English, Portuguese, and Spanish, potentially excluding relevant evidence from other linguistic and cultural contexts.

Finally, no formal methodological appraisal or certainty-of-evidence assessment was undertaken. The findings therefore represent a map of reported interventions and outcomes rather than an estimate of comparative effectiveness.

## 5. Conclusions

The available controlled studies indicate short-term, domain-specific patterns in reported outcomes among people with mild-to-moderate Alzheimer’s disease. Conventional and technology-assisted cognitive training primarily showed signals in cognitive outcomes, whereas reminiscence-based approaches more consistently reported changes in depressive and neuropsychiatric symptoms. However, given the small number of studies, limited sample size, heterogeneous interventions and outcome measures, and restricted long-term follow-up, these findings should not be interpreted as evidence of intervention or comparative effectiveness. Evidence regarding functional transfer, acceptability, comparative effectiveness, and long-term maintenance remains insufficient. Future studies should use larger and more diverse samples, clearly described and replicable interventions, standardized core outcomes, active comparators, and longer follow-up, including evaluation of maintenance or booster strategies.

## Figures and Tables

**Table 1:** Database-specific search strategies and number of records retrieved.

Database	Number of Results
PubMed: (“Alzheimer Disease” [Mesh] OR Alzheimer [Title/Abstract]) AND (“cognitive stimulation” [Title/Abstract]) AND (“Aged” [Mesh] OR elderly [Title/Abstract] OR “older adult” [Title/Abstract])	143
SciELO: Aged AND cognitive stimulation AND Alzheimer	10
LILACS: Aged AND cognitive stimulation AND Alzheimer	23
PEDro: Aged AND cognitive stimulation AND Alzheimer	76
Google Scholar: Aged AND cognitive stimulation AND Alzheimer	100

**Table 2:** Characteristics of the included studies and intervention delivery.

Study	Country/Design/Setting	Participants	Intervention and Delivery Format	Dose and Provider	Comparator/Assessment
Oliveira et al. (2021) [7]	Portugal; open-label pilot randomized controlled trial; residential care homes	17 participants with mild-to-moderate dementia due to Alzheimer’s disease	Technology-assisted cognitive stimulation using a computerized non-immersive virtual-reality program reproducing instrumental activities of daily living; supervised computer-based delivery	12 sessions; 45 min/session; 2 sessions/week; approximately 9 h total; delivered by clinical neuropsychologists	Treatment as usual; baseline and post-intervention neuropsychological assessment
Giovagnoli et al. (2017) [8]	Italy; randomized, controlled, single-blind study; single-centre clinical setting	39 participants with mild-to-moderate Alzheimer’s disease	Conventional cognitive training targeting attention, information processing, executive functions, and memory; group-based, with three participants per group	24 sessions over 12 weeks; 2 sessions/week; 45 min/session; coordinated by a neuropsychologist experienced in dementia and group management	Active music therapy or neuroeducation; assessment at baseline, treatment completion, and 3-month follow-up
Li et al. (2020) [9]	China; single-blind randomized parallel controlled trial; Beijing Geriatric Hospital	90 participants with mild-to-moderate Alzheimer’s disease	Group reminiscence therapy	24 sessions over 12 weeks; 2 sessions/week; 30–45 min/session; provider not clearly reported	Conventional drug treatment and routine care; assessment at baseline, 4, 12, and 24 weeks
Onieva-Zafra et al. (2018) [10]	Spain; controlled quasi-experimental pilot pretest–posttest study; nursing home	19 residents with mild Alzheimer’s disease (intervention n = 9; control n = 10)	Multicomponent music–reminiscence–reality-orientation intervention; group nursing intervention using familiar music, reminiscence, and reality-orientation techniques	16 sessions over 8 weeks; 2 sessions/week; 45 min/session; conducted by a nurse	Standard care/control group; baseline and post-intervention assessment
Cavallo et al. (2016) and Cavallo and Angilletta (2019) [11,12]	Italy; randomized controlled trial with subsequent long-term follow-up; clinical setting	80 participants with early-stage Alzheimer’s disease	Technology-assisted computerized cognitive training targeting memory, attention, executive function, and language; supervised individual computer-based training	36 sessions over 12 weeks; 3 sessions/week; 30 min/session; supervised by a neuropsychologist	Computer-based control activities following the same schedule; assessment at baseline, post-intervention, 6-month follow-up, and subsequent 12-month follow-up

**Table 3:** Reported outcomes, statistical interpretation, and limitations of the included studies.

Study	Outcome Domains	Main Reported Findings and Statistical Interpretation	Follow-Up/Limitations
Oliveira et al. (2021) [7]	Global cognition; executive function; memory; attention; instrumental activities of daily living	A statistically significant group × time interaction was reported for global cognition (MMSE; *p* = 0.042), with significant pre–post improvement in the experimental group (*p* = 0.033) and a large effect size. No statistically significant effect was found for executive function measured with the FAB or for IADL.	Small pilot sample; treatment-as-usual control; no long-term follow-up. The study was not powered to establish definitive effectiveness or clinical meaningfulness.
Giovagnoli et al. (2017) [8]	Initiative; episodic memory; depression; anxiety; social relationships	Initiative significantly improved after cognitive training. Approximately 62% of participants receiving cognitive training achieved a clinically significant improvement in initiative, compared with about 8% after active music therapy and none after neuroeducation. Episodic memory did not significantly improve but remained stable at treatment completion. Mood and social relationships improved across treatment groups, with larger psychosocial changes after active music therapy or neuroeducation.	Initiative and episodic memory declined across groups at the 3-month follow-up. Small sample distributed across three treatment arms.
Li et al. (2020) [9]	Cognition; depression; neuropsychiatric symptoms; activities of daily living	Group reminiscence therapy produced statistically significant improvements in depressive and neuropsychiatric symptoms compared with control care (*p* < 0.05). Depression scores showed significant between-group differences at both treatment completion and 12-week post-treatment follow-up. Clear between-group benefits were not demonstrated for cognition or activities of daily living.	Single-centre study. Benefits were clearest for emotional and neuropsychiatric outcomes rather than cognitive or functional outcomes. Clinical meaningfulness was not separately established.
Onieva-Zafra et al. (2018) [10]	Depression; anxiety	The multicomponent music–reminiscence–reality-orientation intervention produced a statistically significant reduction in depressive symptoms. A statistically significant benefit for anxiety was not clearly demonstrated.	Very small non-randomized pilot sample; no long-term follow-up; findings should be considered preliminary. Clinical meaningfulness was not formally established.
Cavallo et al. (2016) and Cavallo and Angilletta (2019) [11,12]	Short-term and working memory; ecological memory; language comprehension; executive function	Computerized cognitive training produced significant group × time interaction effects for short-term and working memory, ecological memory, language comprehension, and executive-function measures, with the experimental group performing better than controls. These improvements were maintained at 6 months but decreased by the 12-month follow-up.	Effects attenuated over longer follow-up. Transfer of improvements to everyday functioning remained uncertain, and clinical meaningfulness was not formally established.

Note. Statistical significance is reported only when explicitly supported by the original studies. Clinical meaningfulness is distinguished from statistical significance and is reported only when specifically evaluated by the study authors. Given the small samples and heterogeneous outcome measures, findings should not be interpreted as estimates of comparative effectiveness.

## Data Availability

The data charted from the included studies are presented in this article. The completed PRISMA-ScR checklist is provided as Supplementary Material S1. Additional review materials, including the screening log and data-charting form, may be made available by the corresponding author on reasonable request.

## Supplementary Materials

The following supporting information can be downloaded at: https://www.mdpi.com/article/10.3390/brainsci16090944/s1, Supplementary Material S1-PRISMA-ScR Checklist.

## Abbreviations

The following abbreviations are used in this manuscript:

AD	Alzheimer’s disease
IADL	Instrumental activities of daily living
JBI	Joanna Briggs Institute
PRISMA-ScR	Preferred Reporting Items for Systematic Reviews and Meta-Analyses extension for Scoping Reviews
VR	Virtual reality
